# 2,3-Bis(methyl­sulfan­yl)-1,4,5,8-tetra­thia­fulvalene

**DOI:** 10.1107/S1600536810048580

**Published:** 2010-11-27

**Authors:** Ning-Juan Zheng, Bing-Zhu Yin

**Affiliations:** a Key Laboratory of Organism Functional Factors of the Changbai Mountain, Yanbian University, Ministry of Education, Yanji 133002, People’s Republic of China

## Abstract

In the title compound, C_8_H_8_S_6_, the five-membered rings form a dihedral angle of 25.06 (9)°. In the absence of short inter­molecular contacts, the mol­ecules are packed by van der Waals forces in the crystal.

## Related literature

For applications of tetra­thia­fulvalenes, see: Wudl *et al.* (1972 ▶); Jørgensen *et al.* (1994 ▶). For details of the synthesis, see: Fourmingué *et al.* (1993 ▶). For a related structure, see: Hou *et al.* (2010 ▶).
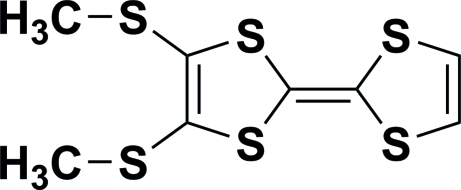


## Experimental

### 

#### Crystal data

C_8_H_8_S_6_
*M* *_r_* = 296.50
Monoclinic, 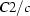

*a* = 19.368 (11) Å
*b* = 7.703 (4) Å
*c* = 17.150 (8) Å
β = 108.59 (2)°
*V* = 2425 (2) Å^3^
*Z* = 8
Mo *K*α radiation
μ = 1.09 mm^−1^
*T* = 291 K
0.12 × 0.10 × 0.09 mm

#### Data collection

Rigaku R-AXIS RAPID diffractometer
Absorption correction: multi-scan (*ABSCOR*; Higashi, 1995 ▶) *T*_min_ = 0.881, *T*_max_ = 0.909
11301 measured reflections
2773 independent reflections
2480 reflections with *I* > 2σ(*I*)
*R*_int_ = 0.025

#### Refinement

*R*[*F* ^2^ > 2σ(*F* ^2^)] = 0.031
*wR*(*F* ^2^) = 0.083
*S* = 1.03
2773 reflections
129 parameters
H-atom parameters constrained
Δρ_max_ = 0.58 e Å^−3^
Δρ_min_ = −0.66 e Å^−3^

### 

Data collection: *RAPID-AUTO* (Rigaku, 1998 ▶); cell refinement: *RAPID-AUTO*; data reduction: *CrystalStructure* (Rigaku/MSC, 2002 ▶); program(s) used to solve structure: *SHELXS97* (Sheldrick, 2008 ▶); program(s) used to refine structure: *SHELXL97* (Sheldrick, 2008 ▶); molecular graphics: *PLATON* (Spek, 2009 ▶); software used to prepare material for publication: *SHELXL97*.

## Supplementary Material

Crystal structure: contains datablocks global, I. DOI: 10.1107/S1600536810048580/ng5069sup1.cif

Structure factors: contains datablocks I. DOI: 10.1107/S1600536810048580/ng5069Isup2.hkl

Additional supplementary materials:  crystallographic information; 3D view; checkCIF report

## Figures and Tables

**Fig. 1.**
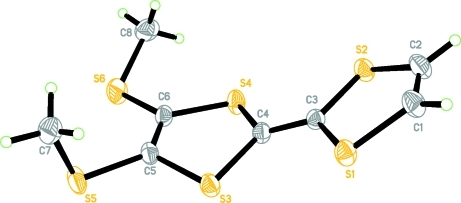
The asymmetric of title compound, with the atom numbering. Displacement ellipsoids of non-H atoms are drawn at the 30% probalility level.

## supplementary crystallographic information

### Comment

Tetrathiafulvalenes (TTFs) have attracted much interest due to their electron-donating ability, which have been used for the synthesis of new organic metals and superconductors (Wudl *et al.* 1972) and recently for supramolecular architectures (Jørgensen *et al.* 1994.). In this paper, we report the crystal structue of the title compound.

The title compound, as shown in Fig. 1, crystallizes in monoclinic system with the space group *C*2/*c*. All bond lengths and angles of the title compound are normal and comparable with those reported for the related structure (Hou *et al.*, 2010). In the crystal, the molecules are packed by van der Waal's forces.

### Experimental

The title compound was prepared according to literature (Fourmingué *et al.*, 1993) and single crystals suitable for X-ray diffraction were prepared by slow evaporation a mixture of dichloromethane and petroleum (60–90 °C) at room temperature.

### Refinement

Carbon-bound H-atoms were placed in calculated positions with C—H = 0.93 or 0.96 Å and were included in the refinement in the riding model with *U*_iso_(H) = 1.2 or 1.5 *U*_eq_(C).

### Crystal data

**Table:**

C_8_H_8_S_6_	*F*(000) = 1216
*M**_r_* = 296.50	*D*_x_ = 1.624 Mg m^−^^3^
Monoclinic, *C*2/*c*	Mo *K*α radiation, λ = 0.71073 Å
Hall symbol: -C 2yc	Cell parameters from 9674 reflections
*a* = 19.368 (11) Å	θ = 3.3–27.6°
*b* = 7.703 (4) Å	µ = 1.09 mm^−^^1^
*c* = 17.150 (8) Å	*T* = 291 K
β = 108.59 (2)°	Block, yellow
*V* = 2425 (2) Å^3^	0.12 × 0.10 × 0.09 mm
*Z* = 8	

### Data collection

**Table:**

Rigaku R-AXIS RAPID diffractometer	2773 independent reflections
Radiation source: fine-focus sealed tube	2480 reflections with *I* > 2σ(*I*)
graphite	*R*_int_ = 0.025
ω scans	θ_max_ = 27.5°, θ_min_ = 3.3°
Absorption correction: multi-scan (*ABSCOR*; Higashi, 1995)	*h* = −25→25
*T*_min_ = 0.881, *T*_max_ = 0.909	*k* = −10→9
11301 measured reflections	*l* = −21→22

### Refinement

**Table:**

Refinement on *F*^2^	Primary atom site location: structure-invariant direct methods
Least-squares matrix: full	Secondary atom site location: difference Fourier map
*R*[*F*^2^ > 2σ(*F*^2^)] = 0.031	Hydrogen site location: inferred from neighbouring sites
*wR*(*F*^2^) = 0.083	H-atom parameters constrained
*S* = 1.03	*w* = 1/[σ^2^(*F*_o_^2^) + (0.043*P*)^2^ + 2.0438*P*] where *P* = (*F*_o_^2^ + 2*F*_c_^2^)/3
2773 reflections	(Δ/σ)_max_ = 0.006
129 parameters	Δρ_max_ = 0.58 e Å^−^^3^
0 restraints	Δρ_min_ = −0.66 e Å^−^^3^

### Special details

**Table:**

Experimental. (See detailed section in the paper)
Geometry. All e.s.d.'s (except the e.s.d. in the dihedral angle between two l.s. planes) are estimated using the full covariance matrix. The cell e.s.d.'s are taken into account individually in the estimation of e.s.d.'s in distances, angles and torsion angles; correlations between e.s.d.'s in cell parameters are only used when they are defined by crystal symmetry. An approximate (isotropic) treatment of cell e.s.d.'s is used for estimating e.s.d.'s involving l.s. planes.
Refinement. Refinement of *F*^2^ against ALL reflections. The weighted *R*-factor *wR* and goodness of fit *S* are based on *F*^2^, conventional *R*-factors *R* are based on *F*, with *F* set to zero for negative *F*^2^. The threshold expression of *F*^2^ > σ(*F*^2^) is used only for calculating *R*-factors(gt) *etc*. and is not relevant to the choice of reflections for refinement. *R*-factors based on *F*^2^ are statistically about twice as large as those based on *F*, and *R*- factors based on ALL data will be even larger.

### Fractional atomic coordinates and isotropic or equivalent isotropic displacement parameters (Å^2^)

**Table:**

	*x*	*y*	*z*	*U*_iso_*/*U*_eq_	
C1	0.02208 (11)	0.7309 (3)	1.15260 (11)	0.0490 (5)	
H1	−0.0004	0.7911	1.1849	0.059*	
C2	0.06543 (12)	0.5980 (3)	1.18273 (11)	0.0494 (5)	
H2	0.0744	0.5615	1.2368	0.059*	
C3	0.07086 (9)	0.6402 (2)	1.03633 (10)	0.0331 (3)	
C4	0.09038 (9)	0.6398 (2)	0.96816 (10)	0.0348 (3)	
C5	0.12943 (10)	0.7609 (2)	0.84888 (10)	0.0375 (4)	
C6	0.17548 (9)	0.6286 (2)	0.87949 (10)	0.0381 (4)	
C7	0.18062 (13)	1.0834 (3)	0.83038 (14)	0.0552 (5)	
H7A	0.1538	1.1248	0.8651	0.083*	
H7B	0.1850	1.1747	0.7941	0.083*	
H7C	0.2283	1.0472	0.8638	0.083*	
C8	0.30945 (12)	0.4634 (3)	0.92717 (15)	0.0608 (6)	
H8A	0.3209	0.5384	0.9743	0.091*	
H8B	0.3531	0.4385	0.9141	0.091*	
H8C	0.2890	0.3571	0.9391	0.091*	
S1	0.00813 (3)	0.79005 (6)	1.05124 (3)	0.04253 (13)	
S2	0.10518 (3)	0.49359 (6)	1.11799 (3)	0.04402 (13)	
S3	0.05498 (2)	0.78415 (6)	0.88578 (3)	0.04151 (13)	
S4	0.15503 (3)	0.49566 (6)	0.95261 (3)	0.04458 (14)	
S5	0.13348 (3)	0.90281 (7)	0.77079 (3)	0.05321 (15)	
S6	0.24524 (3)	0.56800 (10)	0.84168 (4)	0.06234 (18)	

### Atomic displacement parameters (Å^2^)

**Table:**

	*U*^11^	*U*^22^	*U*^33^	*U*^12^	*U*^13^	*U*^23^
C1	0.0601 (12)	0.0557 (12)	0.0353 (9)	0.0035 (9)	0.0211 (8)	−0.0054 (8)
C2	0.0591 (11)	0.0599 (13)	0.0293 (9)	0.0019 (10)	0.0142 (8)	0.0037 (8)
C3	0.0323 (7)	0.0330 (8)	0.0354 (8)	0.0023 (6)	0.0127 (6)	0.0048 (6)
C4	0.0353 (8)	0.0347 (9)	0.0377 (8)	0.0030 (6)	0.0160 (7)	0.0063 (6)
C5	0.0468 (9)	0.0376 (9)	0.0311 (8)	−0.0072 (7)	0.0166 (7)	0.0002 (6)
C6	0.0405 (8)	0.0442 (10)	0.0343 (8)	−0.0055 (7)	0.0185 (7)	−0.0019 (7)
C7	0.0602 (12)	0.0458 (12)	0.0593 (13)	−0.0100 (10)	0.0187 (10)	0.0034 (9)
C8	0.0425 (11)	0.0773 (16)	0.0651 (14)	0.0057 (10)	0.0206 (10)	−0.0037 (12)
S1	0.0492 (3)	0.0424 (3)	0.0420 (2)	0.01172 (19)	0.0229 (2)	0.00695 (18)
S2	0.0462 (3)	0.0476 (3)	0.0400 (2)	0.01051 (19)	0.0161 (2)	0.01361 (18)
S3	0.0447 (2)	0.0423 (3)	0.0404 (2)	0.00839 (19)	0.01765 (19)	0.01208 (18)
S4	0.0482 (3)	0.0449 (3)	0.0505 (3)	0.0142 (2)	0.0295 (2)	0.01503 (19)
S5	0.0789 (4)	0.0510 (3)	0.0320 (2)	−0.0161 (3)	0.0210 (2)	0.00457 (19)
S6	0.0543 (3)	0.0919 (5)	0.0539 (3)	0.0106 (3)	0.0356 (3)	0.0085 (3)

### Geometric parameters (Å, °)

**Table:**

C1—C2	1.321 (3)	C5—S3	1.759 (2)
C1—S1	1.733 (2)	C6—S6	1.7386 (19)
C1—H1	0.9300	C6—S4	1.7591 (18)
C2—S2	1.737 (2)	C7—S5	1.794 (2)
C2—H2	0.9300	C7—H7A	0.9600
C3—C4	1.339 (2)	C7—H7B	0.9600
C3—S1	1.7525 (18)	C7—H7C	0.9600
C3—S2	1.7565 (17)	C8—S6	1.784 (2)
C4—S3	1.7565 (18)	C8—H8A	0.9600
C4—S4	1.7562 (18)	C8—H8B	0.9600
C5—C6	1.346 (3)	C8—H8C	0.9600
C5—S5	1.7498 (18)		
			
C2—C1—S1	118.21 (15)	S5—C7—H7A	109.5
C2—C1—H1	120.9	S5—C7—H7B	109.5
S1—C1—H1	120.9	H7A—C7—H7B	109.5
C1—C2—S2	117.70 (15)	S5—C7—H7C	109.5
C1—C2—H2	121.1	H7A—C7—H7C	109.5
S2—C2—H2	121.1	H7B—C7—H7C	109.5
C4—C3—S1	122.09 (13)	S6—C8—H8A	109.5
C4—C3—S2	123.66 (14)	S6—C8—H8B	109.5
S1—C3—S2	114.26 (9)	H8A—C8—H8B	109.5
C3—C4—S3	123.67 (14)	S6—C8—H8C	109.5
C3—C4—S4	123.23 (13)	H8A—C8—H8C	109.5
S3—C4—S4	113.10 (10)	H8B—C8—H8C	109.5
C6—C5—S5	125.86 (14)	C1—S1—C3	94.57 (9)
C6—C5—S3	117.01 (13)	C2—S2—C3	94.58 (10)
S5—C5—S3	116.99 (11)	C4—S3—C5	94.04 (8)
C5—C6—S6	123.71 (14)	C4—S4—C6	94.24 (9)
C5—C6—S4	116.55 (14)	C5—S5—C7	100.79 (10)
S6—C6—S4	119.21 (11)	C6—S6—C8	103.63 (10)
